# Stochastic Delay Accelerates Signaling in Gene Networks

**DOI:** 10.1371/journal.pcbi.1002264

**Published:** 2011-11-10

**Authors:** Krešimir Josić, José Manuel López, William Ott, LieJune Shiau, Matthew R. Bennett

**Affiliations:** 1 Department of Mathematics, University of Houston, Houston, Texas, United States of America; 2 Department of Biology and Biochemistry, University of Houston, Houston, Texas, United States of America; 3 Department of Mathematics, University of Houston, Clear Lake, Texas, United States of America; 4 Department of Biochemistry & Cell Biology, Rice University, Houston, Texas, United States of America; 5 Institute of Biosciences & Bioengineering, Rice University, Houston, Texas, United States of America; North Carolina State University, United States of America

## Abstract

The creation of protein from DNA is a dynamic process consisting of numerous reactions, such as transcription, translation and protein folding. Each of these reactions is further comprised of hundreds or thousands of sub-steps that must be completed before a protein is fully mature. Consequently, the time it takes to create a single protein depends on the number of steps in the reaction chain and the nature of each step. One way to account for these reactions in models of gene regulatory networks is to incorporate dynamical delay. However, the stochastic nature of the reactions necessary to produce protein leads to a waiting time that is randomly distributed. Here, we use queueing theory to examine the effects of such distributed delay on the propagation of information through transcriptionally regulated genetic networks. In an analytically tractable model we find that increasing the randomness in protein production delay can increase signaling speed in transcriptional networks. The effect is confirmed in stochastic simulations, and we demonstrate its impact in several common transcriptional motifs. In particular, we show that in feedforward loops signaling time and magnitude are significantly affected by distributed delay. In addition, delay has previously been shown to cause stable oscillations in circuits with negative feedback. We show that the period and the amplitude of the oscillations monotonically decrease as the variability of the delay time increases.

## Author Summary

Delay in gene regulatory networks often arises from the numerous sequential reactions necessary to create fully functional protein from DNA. While the molecular mechanisms behind protein production and maturation are known, it is still unknown to what extent the resulting delay affects signaling in transcriptional networks. In contrast to previous studies that have examined the consequences of fixed delay in gene networks, here we investigate how the variability of the delay time influences the resulting dynamics. The exact distribution of “transcriptional delay” is still unknown, and most likely greatly depends on both intrinsic and extrinsic factors. Nevertheless, we are able to deduce specific effects of distributed delay on transcriptional signaling that are independent of the underlying distribution. We find that the time it takes for a gene encoding a transcription factor to signal its downstream target decreases as the delay variability increases. We use queueing theory to derive a simple relationship describing this result, and use stochastic simulations to confirm it. The consequences of distributed delay for several common transcriptional motifs are also discussed.

## Introduction

Gene regulation forms a basis for cellular decision-making processes and transcriptional signaling is one way in which cells can modulate gene expression patterns [1]. The intricate networks of transcription factors and their targets are of intense interest to theorists because it is hoped that topological similarities between networks will reveal functional parallels [2]. Models of gene regulatory networks have taken many forms, ranging from simplified Boolean networks [3], [4], to full-scale, stochastic descriptions simulated using Gillespie's algorithm [5].

The majority of models, however, are systems of nonlinear ordinary differential equations (ODEs). Yet, because of the complexity of protein production, ODE models of transcriptional networks are at best heuristic reductions of the true system, and often fail to capture many aspects of network dynamics. Many ignored reactions, like oligomerization of transcription factors or enzyme-substrate binding, occur at much faster timescales than reactions such as transcription and degradation of proteins. Reduced models are frequently obtained by eliminating these fast reactions [6]–[9]. Unfortunately, even when such reductions are done correctly, problems might still exist. For instance, if within the reaction network there exists a linear (or approximately linear) sequence of reactions, the resulting dynamics can appear to be delayed. This type of behavior has long been known to exist in gene regulatory networks [10].

Delay differential equations (DDEs) have been used as an alternative to ODE models to address this problem. In protein production, one can think of delay as resulting from the sequential assembly of first mRNA and then protein [10]–[12]. Delay can qualitatively alter the local stability of genetic regulatory network models [13] as well as their dynamics, especially in those containing feedback. For instance, delay can lead to oscillations in models of transcriptional negative feedback [11], [14]–[18], and experimental evidence suggests that robust oscillations in simple synthetic networks are due to transcriptional delay [19], [20].

Protein production delay times are difficult to measure in live cells, though recent work has shown that the time it takes for transcription to occur in yeast can be on the order of minutes and is highly variable [21]. Still, transcriptional delay is thought to be important in a host of naturally occurring gene networks. For instance, mathematical models suggest that circadian oscillations are governed by delayed negative feedback systems [22], [23], and this was experimentally shown to be true in mammalian cells [24]. Delay appears to play a role in cell cycle control [25], [26], apoptosis induction by the p53 network [27], and the response of the 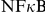 network [15]. Delay can also affect the stochastic nature of gene expression, and the relation between the two can be subtle and complex [28]–[31].

In this study, we examine the consequences of randomly distributed delay on simple gene regulatory networks: We assume that the delay time for protein production, 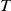, is not constant but instead a random variable. If 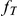 denotes the probability density function (PDF) of 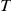, this situation can be described deterministically by an integro-delay differential equation [32] of the form
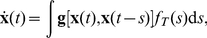
(1)where 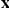 is a positive definite state vector of protein concentrations, and 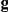 is a vector function representing the production and degradation rates of the proteins. Note that processes that do not require protein synthesis (like dilution and degradation) will depend on the instantaneous, rather than the delayed, state of the system. Therefore 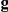 is in general a function of both the present and past state of the system.

Equation (1) only holds in the limit of large protein numbers [32]. As protein numbers approach zero, the stochasticity associated with chemical interactions becomes non-negligible. Here, we address this issue by expanding on Eq. (1) using an exact stochastic algorithm that takes into account variability within the delay time [32]. We further use a queueing theory approach to examine how this variability affects timing in signaling cascades. We find that when the mean of the delay time is fixed, increased delay variability accelerates downstream signaling. Noise can thus increase signaling speed in gene networks. In addition, we find that in simple transcriptional networks containing feed-forward or feedback loops the variability in the delay time nontrivially affects network dynamics.

Queueing theory has recently been used to understand the behavior of genetic networks [33]–[35]. Here we are mainly interested in dynamical phenomena to which the theory of queues in equilibrium used in previous studies cannot be applied. As we explain below, gene networks can be modeled as thresholded queueing systems: Proteins exiting one queue do not enter another queue, as would be the case in typical queueing networks. Rather, they modulate the *rate* at which transcription is initiated, and thus affect the *rate* at which proteins enter other queues.

## Results

### Distributed delay in protein production

**Figure 1:**
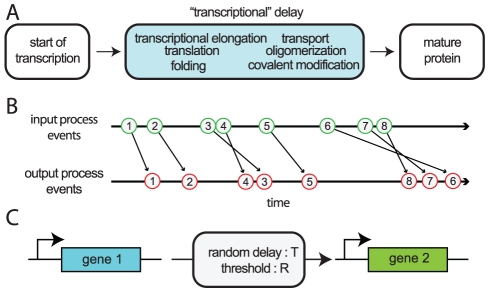
The origin of delay in transcriptional regulation. (A) Numerous reactions must occur between the time that transcription starts and when the resulting protein molecule is fully formed and mature. Though we call this phenomenon “transcriptional” delay, there are many reactions after transcription (such as translation) which contribute to the overall delay. (B) The creation of multiple proteins can be thought of as a queueing process. Nascent proteins enter the queue (an input event) and emerge fully matured (an output event) some time later depending on the distribution of delay times. Because the delay is random, it is possible that the order of proteins entering the queue is not preserved upon exit. (C) In a transcriptionally regulated signaling process the time it takes for changes in the expression of gene 1 to propagate to gene 2 depends on both the distribution of delay times, 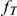, and the number of transcription factors needed to overcome the threshold of gene 2, 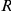.

The transcription of genetic material into mRNA and its subsequent translation into protein involves potentially hundreds or thousands of biochemical reactions. Hence, detailed models of these processes are prohibitively complex. When simulating genetic circuits it is frequently assumed that gene expression instantaneously results in fully formed proteins. However, each step in the chain of reactions leading from transcription initiation to a folded protein takes time (Figure 1). Models that do not incorporate the resulting delay may not accurately capture the dynamical behavior of genetic circuits [17]. While earlier models have included either fixed or distributed delay [32], [36], [37], here we examine specifically the *effects* of delay variability on transcriptional signaling.

In one recent study, Bel *et al.* studied completion time distributions associated with Markov chains modeling linear chemical reaction pathways [38]. Using rigorous analysis and numerical simulations they show that, if the number of reactions is large, completion time distributions for an idealized class of models exhibit a sharp transition in the coefficient of variation (CV, defined as the standard deviation divided by the mean of the distribution), going from near 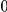 (indicating a nearly deterministic completion time) to near 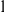 (indicating an exponentially distributed completion time) as system bias moves from forward to reverse.

However, it is possible, and perhaps likely, that the limiting distributions described by Bel *et al.* do not provide good approximations for protein production. For instance, when the number of rate limiting reactions is small, but greater than one, the distribution of delay times can be more complex. Moreover, linear reaction pathways only represent one possible and necessarily simplified reaction scheme. Protein production involves many reaction types that are nonlinear and/or reversible, each of which is influenced by intrinsic and extrinsic noise [39], and these reactions may impact the delay time distribution in complicated ways. Therefore, we do not try to derive the actual shape of 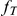, but examine the effects its statistical properties have on transcriptional signaling. To do this, we represent protein production as a delayed reaction of the form
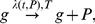
(2)where 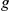 is the gene, and transcription is initiated at rate 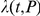, which can depend explicitly on both time and protein number, 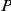. After initiation, it takes a random time, 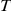, for a protein to be formed. Note that the presence of time delay implies that scheme (2) defines a non-Markovian process. Such processes can be simulated exactly using an extension of the Gillespie algorithm (See Methods and [28], [32]).

If the biochemical reaction pathway that leads to functional protein is known and relatively simple, direct stochastic simulation of every step in the network is preferable to simulation based on scheme (2). From the point of view of multi-scale modeling, however, paradigm (2) is useful when the biochemical reaction network is either extremely complex or poorly mapped, since one needs to know only the statistical properties of 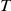.

### Protein formation as a queueing system

In the setting of scheme (2), first assume that 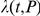 does not depend on 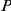, and protein formation is initiated according to a memoryless process with rate 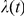. A fully formed protein enters the population a random time 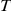 after the initiation of protein formation. We assume that the molecules do not interact while forming; that is, the formation of one protein does not affect that of another. Each protein therefore emerges from an independent reaction channel after a random time. This process is equivalent to an 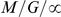 queue [40], where 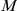 indicates a memoryless source (transcription initiation), 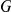 a general service time distribution (delay time distribution), and 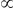 refers to the number of service channels.

In our model, the order in which initiation events enter a queue is not necessarily preserved. As Figure 1 (B) illustrates, it is possible for the initiation order to be permuted upon exit [32]. The assumption that proteins can “skip ahead” complicates the analysis of transient dynamics of such queues, and is essential in much of the following. While there are steps where such skipping can occur (such as protein folding), there are others for which it cannot. For instance, it is unlikely that one RNA polymerase can skip ahead of another – and similarly for ribosomes during translation off of the same transcript. Therefore, protein skipping may be more relevant in eukaryotes, where transcription and translation must occur separately, than prokaryotes, where they may occur simultaneously. However, if there is more than one copy of the gene (which is common for plasmid-based synthetic gene networks in *E. coli*), or more than one transcript, some skipping is likely occur. Therefore it is likely that the full results that follow are more relevant for genes of copy number greater than one.

### Downstream transcriptional signaling

One purpose of transcription factors is to propagate signals to downstream target genes. Determining the dynamics and stochasticity of these signaling cascades is of both theoretical and experimental interest [41], [42]. Therefore, we first examine the impact that distributed delay has on simple downstream signaling. Consider the situation depicted in Figure 1 (C), in which the product of the first gene regulates the transcription of a second gene. Using the same nomenclature as in scheme (2) we write
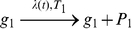
(3a)

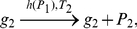
(3b)where 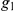 and 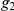 are the copy numbers of the upstream and downstream genes, 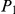 and 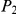 are the number of functional proteins of each type, and 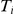 is the random delay time of gene 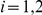. The transcription rate of gene 2 depends on 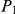 and is given by a Hill function 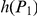. We consider the case in which 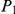 activates 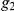 (depicted in Figure 1) and the case in which 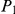 represses 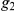.

We now ask: If 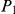 starts at zero and gene 1 is suddenly turned on, how long does it take until the signal is detected by gene 2? In other words, assume 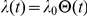, where 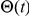 is the Heaviside step function. At what time 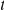 does 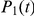 reach a level that is detectable by gene 2? In order to make the problem tractable, we assume that the Hill function is steep and switch-like, so that we can make the approximation
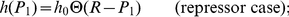
(4a)

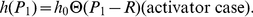
(4b)Here 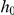 is the maximum transcriptional initiation rate of 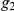 and 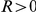 is the threshold value of the Hill function, *i.e.* the number of molecules of 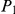 needed for half repression (or half activation) of gene 2. The second gene therefore becomes repressed (or activated) at the time 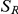 at which 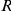 copies of protein 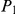 have been fully formed.

We first examine reaction (3a). Assume that at time 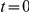 there are no proteins in the system. Let 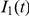 denote the number of transcription initiation events that have occurred by time 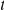 (the arrival process of the queueing system), 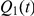 the number of proteins being formed at time 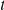 (the size of the queue at time 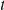), and 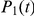 the number of functional proteins that have been completed by time 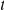 (the exit process of the queueing system). Since the arrival process is memoryless, 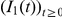 is a Poisson process with constant rate 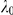 for 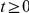. Hence, the expected value of 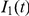 is 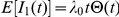.

The exit process, *i.e.* the number of fully functional proteins that have emerged from the queue, 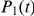, is a nonhomogenous Poisson process with time-dependent rate 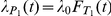, where 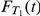 is the cumulative distribution function (CDF) of the delay time 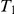. It then follows that
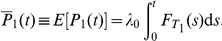



Inactivation (or activation) of gene 2 occurs when enough protein 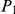 has accumulated to trigger a transcriptional change, according to Eq. (4a) or (4b). In other words, the random time it takes for the signal to propagate, 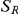, is given by 
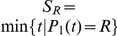
. Trivially, 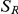 changes by an amount identical to a change in the mean of the delay distribution. To examine the effects of randomness in delay on the signaling time, we therefore keep the mean of the delay distribution fixed, 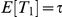, and vary 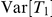.

The probability density function of 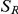 is given by (See Methods)
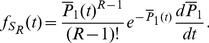
(5)


Consequently, the mean and variance of the time it takes for the original signal to propagate to the downstream gene can be written as:
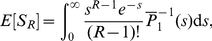
(6)

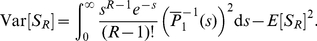
(7)


**Figure 2:**
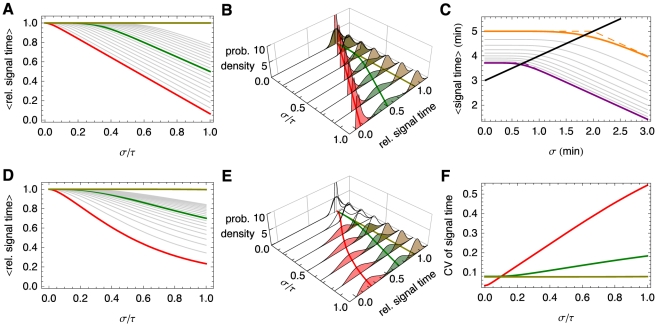
The effects of distributed delay on transcriptional signaling. (A) For the simplified symmetric distribution where the delay takes values 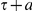 and 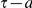 with equal probability, the mean signaling time decreases with increasing variability in delay time, Eq. (8). Shown are the signaling times (normalized by the time at 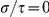), versus CV of the delay time for signaling threshold values from 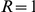 (red), through 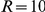 (green) to 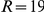 in steps of 1. Here 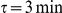 and 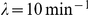. When 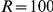 (brown) increasing randomness in delay time has little effect on the mean. (B) Same as panel (A) but with the probability distribution, 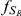, for different values of 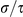. (C) The transition from the small 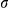 regime to the large 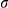 regime occurs when 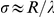. Here we fix 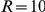 and between the different curves vary 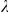 from 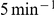 (magenta) to 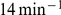 (orange) in steps of 1. Dashed lines show the asymptotic approximations, Eqs. (9) and (10), which meet at the black line. Panels (D) and (E) are equivalent to panels (A) and (B), with 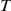 following a gamma distribution, 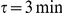, and 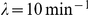. (F) The coefficient of variation of the signaling time, 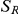, as a function of 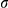.

To gain insight into the behaviors of Eqs. (6) and (7), we first examine a representative, analytically tractable example. Assume that the delay time can take on 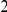 discrete values, 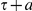 and 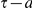 with equal probability. In this case,
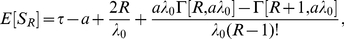
(8)where 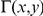 is the upper incomplete gamma function. Expanding for small 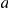, we obtain (See Methods)
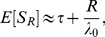
(9)which is the deterministic limit. The first term is the mean delay time and the second is the average time to initiate 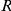 proteins at rate 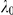. A similar expansion for fixed 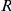 and large 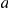 gives (see panel (c) in Figure 2)
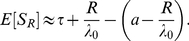
(10)


It follows that for larger delay variability, the mean signaling time *decreases* with delay variability (See Figure 2 (A)). Indeed, Eqs. (9) and (10) form the asymptotic boundaries for the mean signaling time. The intersection of the two asymptotes at 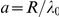, gives an estimate of when the behavior of the system changes from the deterministic limit (for 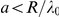) to a regime in which increasing the variability decreases the mean signaling time (for 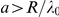). It follows that the deterministic approximation given by Eq. (9) is valid in an increasing range, as 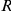 grows (See Figure 2 (C)). Indeed, an asymptotic analysis of Eq. (8) shows that the corrections to Eq. (9) are approximately of size 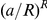, and therefore rapidly decrease with 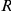 (See Methods).

The bottom row of Figure 2 shows that these observations hold more generally: When 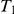 is gamma distributed the mean time to produce 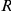 proteins, 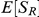, is very sensitive to randomness in delay time, but only when 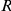 is small to intermediate. As expected, the densities of the times to produce 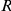 proteins, 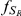, are approximately normal and independent of the delay distribution when 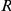 is large (Middle panels of Figure 2).

We therefore expect that for each fixed threshold 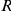, 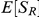 is a decreasing function of the standard deviation 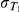 of the delay. We have proved this to be true for symmetric delay distributions (See Methods). Intuitively, this is due to the fact that the order in which proteins enter the queue is not the same as the order in which they exit. Proteins that enter the queue before the 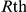 protein, but exit after the 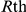 protein increase 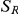, while the opposite is true for proteins that enter the queue after the 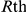 protein, and exit before it. Since only finitely many proteins enter the queue before the 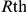 protein, while infinitely many enter after it, the balance favors a decrease in the mean signaling time. Moreover, as delay variability increases, interchanges in exit order become more likely, and this effect becomes more pronounced. We outline the analytical argument: For each fixed time 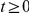, 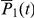 is an increasing function of 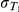, hence 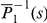 is decreasing function of 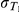 for all 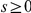. Referring to Eq. (6), this implies that 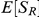 is a decreasing function of 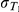 in the symmetric case.

In sum, mean signaling times decrease as delay variability increases (with fixed mean delay). This effect is most significant for small to moderate thresholds. We note that the decrease in mean signaling time phenomenon depends on a sufficient number of proteins entering the queue. If transcription is only active long enough for less than 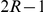 proteins to be initiated, then mean signaling time will actually *increase* as delay variability increases. This phenomenon is explained in the subsection of the Methods section that analyzes repressor switches.

### Example: Feedforward loops

**Figure 3:**
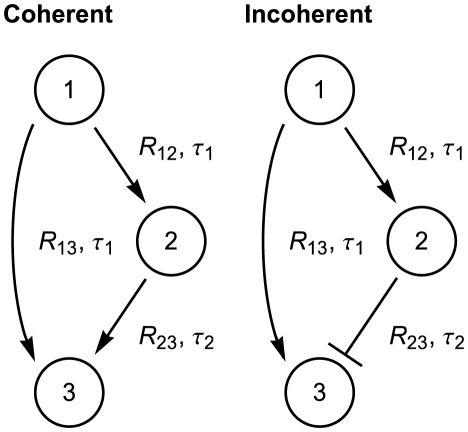
Network schematics for the coherent and incoherent feedforward loops. Each pathway in the networks has an associated signaling threshold (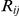) and mean delay time (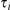). The random time between the initiation of transcription of gene 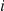 to the full formation of a total of 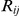 proteins 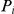 is denoted 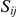, which is an implicit function of 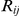.

Using the above results, we now examine more complicated transcriptional signaling networks. In particular, we turn to two common feedforward loops - the type 1 coherent and the type 1 incoherent feedforward loops (FFL) [43], shown in Figure 3. Each of these networks is a transcriptional cascade resulting in the specific response of the output, gene 3. The coherent FFL generally acts as a delayed response network, while the incoherent FFL has various possible responses, such as pulsatile response [43], response time acceleration [44], and fold-change detection [45].

To examine the effect of distributed delay on these networks we assume that at 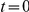 gene 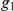 starts transcription of protein 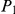 at rate 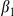, *i.e* 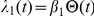. The second gene, 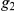, starts transcription after 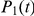 reaches the threshold 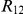, so that 
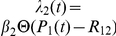
. For the coherent FFL, we assume that the promoter of gene 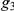 acts as an AND gate so that 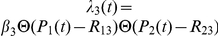. We further assume that the promoter of 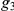 in the incoherent FFL is active only in the presence of 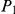 and absence of 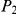, so that we may write 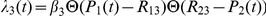.

The signaling time between any two nodes 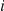 and 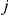 within the network, *i.e.* the random time between the initiation of transcription of gene 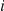 to the formation of a total of 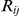 proteins 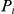 is denoted 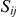. For each of the three pathways, the PDF of the signaling time is given by Eq. (5). In addition, because the random times 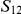 and 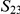 are additive (as are their variances), we can directly calculate the time at which 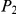 reaches the threshold of gene 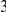 as 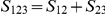. Therefore, the random time at which the coherent FFL turns on is simply given by 
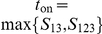
. Because 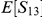 and 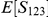 are decreasing functions of the delay variability, it can be expected that so is 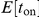.

**Figure 4:**
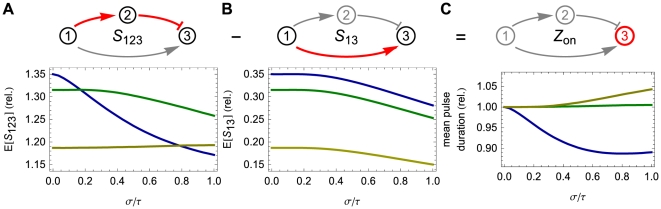
Distributed delay can either increase of decrease pulse duration in an incoherent feedforward loop. (A) *Top*: The longer pathway consists of the sum of two shorter pathways: 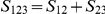. (A) *Bottom*: The expected value of signaling time as a function of the relative standard deviation of the delay time. (B) *Top*: The shorter pathway is simply the signaling of the first gene to the third. (B) *Bottom*: Expected signaling time, 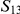. (C) *Top*: The output pulse is determined by the amount of time gene 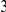 is actively transcribing. This time is simply the difference of the longer path duration (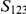) and the shorter path duration (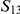). (C) *Bottom*: Depending on the thresholds 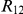, 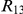, and 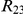, the expected pulse duration can either increase or decrease as a function of the delay variability. In each of the three plots, the data on the vertical axis are presented relative to the mean pulse duration at 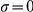. Here, the colored lines correspond to 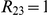 (blue), 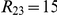 (green), and 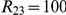 (brown), while 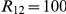, 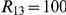. In addition, the protein degradation rates are each 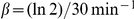, all delays are gamma distributed with mean 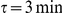.

In contrast to the coherent FFL, the dynamics of the pulse generating incoherent FFL are less trivial. Since the repressor (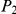) overrides the activator (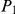), assuming 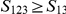 transcription of 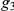 turns on at time 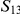 and turns off at time 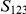, generating a pulse of duration 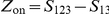. Note that 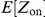 can *increase or decrease* as a function of the standard deviation 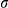 of the delay (see Figure 4 where 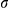 was equal for all pathways).

To see this, write 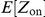 as follows:
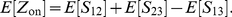
(11)Each of the terms on the right side of Eq. (11) is the expected signaling time of a single gene (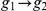, 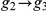, and 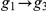, respectively). Consequently, 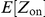 depends on 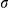 as a linear combination of 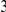 expected signaling time curves of the type pictured in Figure 2. The shapes of these signaling time curves determine the behavior of 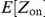 as a function of 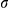. Figure 4 shows that the behavior of the duration of the transcriptional pulse as a function of the delay variability depends on the values of each threshold within the network.

### The delayed negative feedback oscillator

These observations can also be extended to networks with recurrent architectures. For instance, consider the transcriptional delayed negative feedback circuit [17], which can be described using an extension of scheme (2):
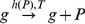
(12a)

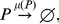
(12b)where 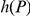 is a decreasing Hill function (i.e. 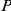 represses its own production) and 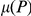 is the degradation rate due to dilution and proteolysis. Mather *et al.* examined the oscillations produced by systems of the type described by scheme (12) when the delay 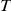 is nonrandom (degrade and fire oscillators) [17]. Starting with no proteins, 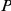 is produced at a rate governed by the Hill function 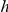. When the level of 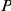 exceeds the midpoint of the Hill function, gene 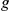 effectively shuts down. The proteins remaining in the queue exit, producing a spike, after which degradation diminishes 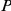. When the protein level drops sufficiently, reaction (12a) reactivates and production of 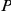 resumes, commencing another oscillation cycle. Note that this circuit will not oscillate without delay.

**Figure 5:**
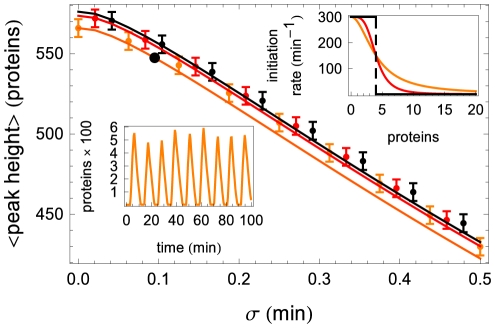
Distributed delay in the delayed negative feedback oscillator. Shown are the analytically predicted (solid lines) and numerically obtained (symbols with standard deviation error bars) mean peak heights of the negative feedback oscillator with Hill coefficients of 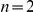 (orange), 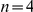 (red), and 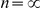 (i.e. step function, black). The top inset shows the shape of the Hill function for the three values of 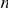, with colors matching those in the main figure. The lower inset shows one realization of the oscillator at parameter values corresponding to the large black circle on the orange (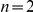) curve of the main figure. The average and the standard deviation of the peak heights were calculated from stochastic simulations of 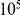 oscillations. Here 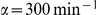, 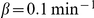, 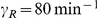, 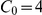 and 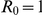.

As a result during each oscillation the gene is turned on until its own signal reaches itself, at which time the gene is turned off [17]. Therefore, the peak height of one oscillation is determined by the length of time the gene was in the “ON” state. Since that time is determined by the gene's signaling time, our theory predicts that the mean peak height of the oscillations will decrease as the variability in the delay time increases. Indeed, this is exactly what our stochastic simulations show in Figure 5. This is consistent with the fact that the negative feedback circuit is dynamically similar to the 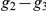 sub-circuit within the incoherent FFL. Here we explicitly used a gamma-distributed delay time with mean 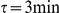, 
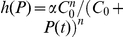
 and 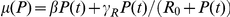.

We can use our theory to predict the change in the peak height of the oscillator as a function of 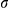. For a delay that is gamma-distributed, the change in signaling time as a function of 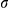 can be written as
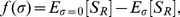
(13)where 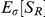 is given by Eq. (6) and 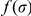 is the reduction in the expected signaling time. If we assume that the amount of time that protein is produced during a burst in the delayed negative feedback oscillator is also reduced by this amount, then it is possible to predict the change in the peak height accordingly. To a first approximation, if the promoter is in the “ON” state for a time that is 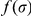 less, then a total of 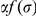 less protein will be produced. Therefore we can write the expected peak height of the oscillator as
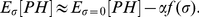
(14)


However, due to degradation, Eq. (14) overestimates the correction to the peak height. Due to exponential degradation, only a fraction 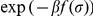 of the lost protein would have made it through to the peak. Also, the duration of enzymatic decay is also reduced by a time 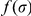. Therefore, if we assume that the enzymatic decay reaction is saturated, we need to add an amount 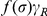 to Eq. (14). This gives us a more accurate prediction of the mean peak height as
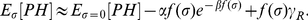
(15)


Figure 5 shows that this approximation works well, even for a Hill coefficient as low as 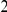.

## Discussion

The existence of delay in the production of protein has been known of for some time. For many systems its presence does not seriously impact performance. For example, the existence of fixed points in simple downstream regulatory networks without feedback is unaffected by delay. Delay is important if the timing of signal propagation impacts the function of the network. Delay can also change a network's dynamics. In networks with feedback, for instance, delay can result in bifurcations that are not present in the corresponding non-delayed system. The delayed negative feedback oscillator is a prime example [17]. Moreover, while the effect of delay in a single reaction may be small, it is cumulative and linearly additive in directed lines.

The intrinsic stochasticity of the reactions that create mature protein make some variation in delay time inevitable. However, we do not yet know the exact nature of this variability or the functional form of the probability density function 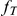. To further complicate matters, there may exist a substantial amount of extrinsic variability in the delay time – the statistics of the PDF may vary from cell to cell.

We focused on the transient dynamics of 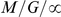 queues in order to demonstrate the effects of distributed delay in a tractable setting. However, as mentioned earlier, 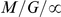 queues may not always be a good model for protein production. For genes with low copy number or few available transcripts queues with 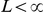 service channels (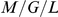 queues) may provide a better description. For eukaryotic systems models in which transcription and translation are decoupled into separate queues may also be relevant. In addition, as protein production rates are often coupled with extrinsic factors such as growth rate and cell cycle phase, 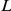 may depend on time and on the state of the system.

The complexity of biochemical reaction networks suggests the use of networks of queues [46], and sources could be toggled on and off by other components of a reaction network. Even protein production from a single transcript may be more accurately described by a sequence of 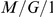 queues with each codon as one in a chain of service stations. In such a model ribosomes move from one codon station to the next, and are not able to skip ahead. Such models will be considered in future studies.

One further complication occurs if the burstiness of the promoter is large [47]. In the above analysis, we assumed that the initiation events of proteins were exponentially distributed in time. Since this is not necessarily the case due to the burstiness of promoters, some limits need to be put on the usefulness of the above results. Equations (9) and (10) suggest that the transition to accelerated behavior occurs when
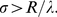
(16)


One can think of 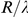 as the average time, 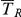, it takes to initiate 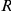 proteins, and rewrite the boundary as 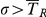. One can then assume that if the burstiness of the initiation events is not large, *i.e.* that the mean burst size is less than the signal threshold, then it does not matter what the distribution of initiation events is. In other words, as long as approximately 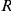 proteins are initiated in the time 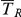, and the variance of that number is not large, then Eq. (16) still holds.

## Methods

### Signaling time distributions associated with a single gene via queueing theory

#### Preliminary information

We first derive the signaling time distributions for a single gene that is modeled by an 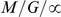 queue. An 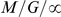 queue is a queueing system consisting of a memoryless arrival process (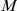) and infinitely many service channels (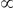). The service time distribution is general (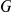) and there exists no maximal system size. Let

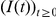
 denote the input (arrival) process,

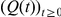
 denote the queue size process, and

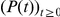
 denote the departure (completion) process.

Thus 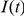, 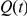, and 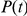 are the numbers of proteins that have entered the queue, are in the queue, and have departed the queue, respectively, at time 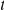. Note that 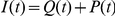 for all 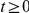. Suppose that 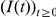 is a nonhomogeneous Poisson process with rate function 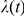. Let 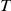 denote the (random) service time and let 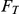 denote the cumulative distribution function (CDF) of 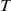. This is the amount of time that a protein spends in the queue after entering. If the distribution of 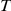 is absolutely continuous, let 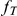 denote the probability density function (PDF) of 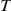. For 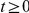, define
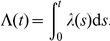
Notice that 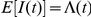 for all 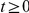.

#### Proposition

(transient distributions; see *e.g.* [40]) *Let* 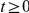 *. The random variables* 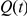 *and* 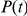 *are Poisson with means*

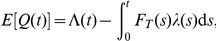


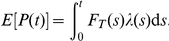



#### Signaling time distributions

Let 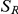 denote the (random) first time at which 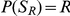, i.e. 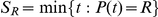. We rescale time so that the rescaled completion process is a homogeneous Poisson process with rate 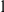. For 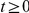 define
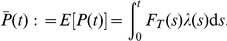



Define the rescaled departure process 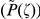 by 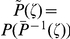. Let 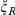 denote the (random) time at which 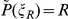. The random time 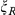 has a gamma distribution with PDF
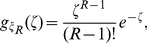
(17)so 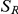 has PDF
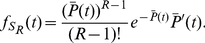
(18)


Computing the expectation of 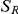, we have
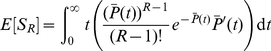
(19a)

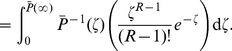
(19b)


We now show that if 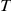 is symmetrically distributed about its mean and 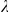 is a constant function, then for every fixed value of 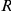, increasing the standard deviation 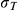 of 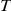 *decreases* the expected signaling time.

#### Proposition

*Assume that* 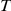 *is symmetrically distributed about its mean and that* 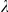 *is a constant function. Let* 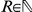 *. The function* 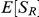 *is a decreasing function of* 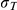 *.*

#### Proof

Suppose that 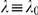. In light of (19b), it suffices to show that for every fixed 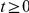, 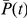 is an increasing function of 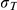. We write 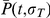 and 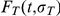 to explicitly indicate the dependence of 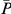 and 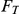 on 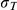 as well as 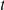. Fix 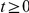 and let 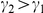. Define 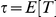. For every 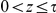, we have
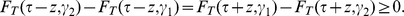
Therefore, if 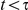, we have
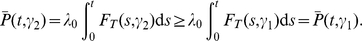



If 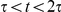, we have
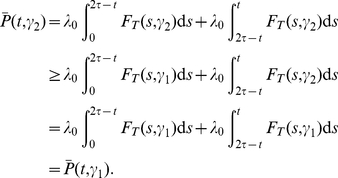
(20)Finally, if 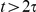, then the inequality 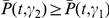 follows from computation (20) and the fact that for 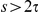, 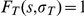 for all relevant values of 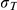.

#### Expected value of 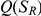


Computing the expectation of 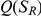, we have
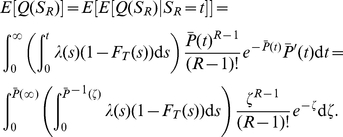



#### Example - Bernoulli delay distributions

Suppose that the rate function of the input process is constant and equal to 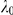, and 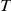 is a Bernoulli random variable described by the probability measure
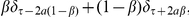
where 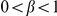. We begin by computing 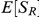.

Let 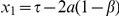 and 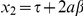. The CDF of 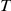 is given by
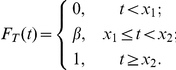



For 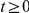 we have
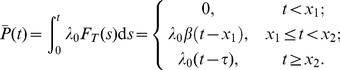



The signaling time 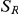 has PDF
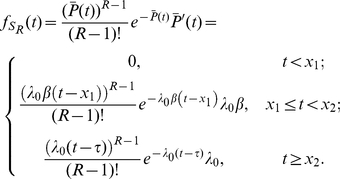



We compute 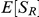 using (19b). The inverse of 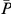 is defined only for 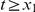, thus
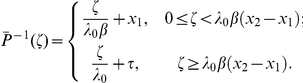



Substituting for 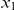 and 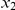 yields
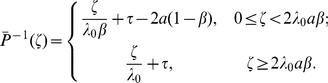



Using (17) and (19b), we have
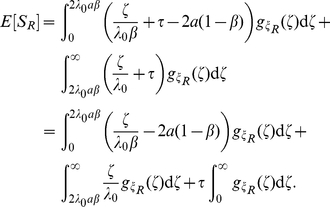
Adding and subtracting 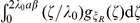 gives
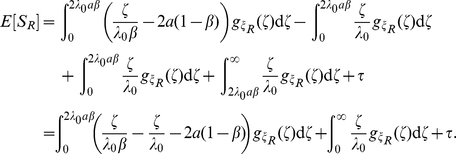
Using 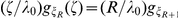, we have
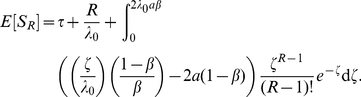
(21)


Finally, we express (21) using gamma functions:
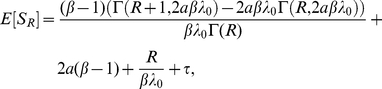
(22)where 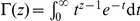 and 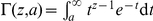.

We now examine the asymptotics of 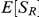 in the 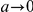 limit. The first and second partial derivatives of 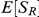 with respect to 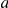 are given by
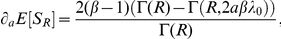


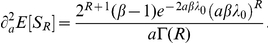



Expanding 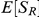 for small values of 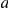 gives
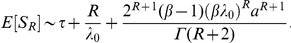



Using the Stirling approximation 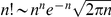 we therefore obtain
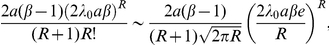
and therefore
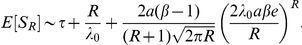



In particular, for 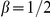 we have
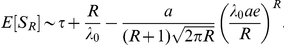



In this case the correction to the deterministic limit is of order 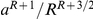.

**Figure 6:**
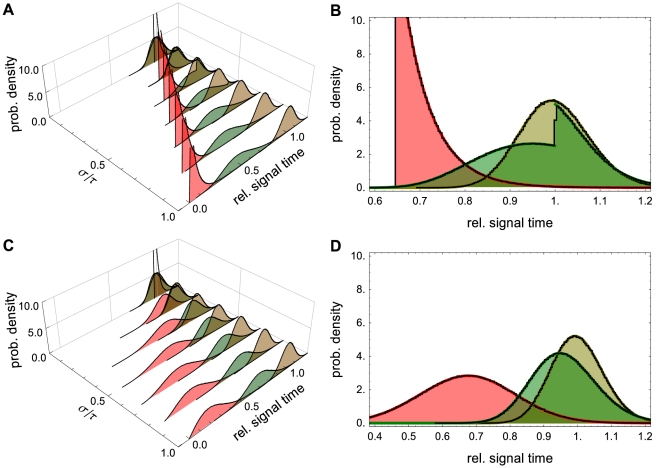
The effects of distributed delay on transcriptional signaling. (A) PDFs for the signaling time using the delay distribution 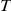 from Example with 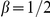. The PDFs in red correspond to signal threshold value 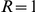, green to 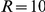 and brown to 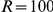. Here 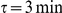 and 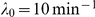. (B) A 2D view of panel (A) with 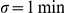. Solid lines show analytical results which are nearly indistinguishable from those obtained through stochastic simulation (black lines). Note that the discontinuity in the green curve is due to the discrete nature of the Bernoulli delay distribution. The CDF, 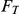, has jump discontinuities that, in light of Eq. (18), produce jump discontinuities in the signaling time PDF. The discontinuity is apparent in both the theoretical prediction (green line) and the stochastic simulations (black line). Panels (C) and (D) are equivalent to panels (A) and (B) with 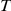 following a gamma distribution. The PDFs were discretized over 200 bins using 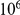 trials.

We obtain linear large 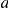 asymptotics by noting that the first term on the right side of (22) vanishes in the 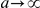 limit:
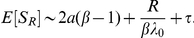



Figure 6 shows a comparison between these analytical results and stochastic simulations.

#### Example 2- Normal delay distributions

Suppose that the rate function of the input process is constant and equal to 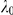. Suppose that 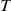 is a normal random variable with mean 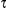 and standard deviation 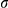.

The CDF of 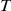 is given by
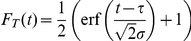
where erf is the error function. For 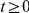 we have
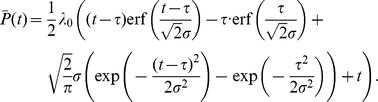



Expanding 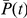 about 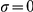 we obtain
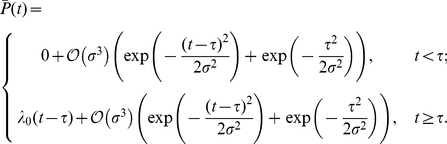



Note that the corrections to the first terms in the expansions are exponentially small in 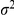 in both regimes. We denote by 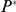 the approximation for 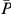 which omits terms exponentially small in 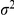. The signaling time PDF of 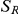 can then be approximated by
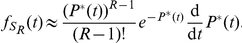



Using (19a), we have
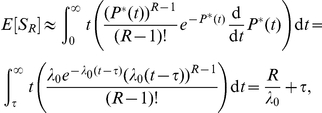
which is again correct up to terms exponentially small in 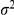.

### Feed-forward network architectures

#### Feed-forward switches

Consider a network of two 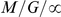 queues with input processes 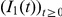 and 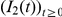, queue size processes 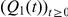 and 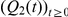, and departure processes 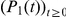 and 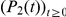. Let 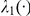 and 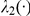 denote the input rate functions of queues 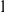 and 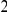, respectively. Queueing system 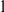 evolves independently of queueing system 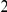 and acts as a switch: at a time which depends on the exit process of the first system, the input process 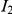 switches on (activator switch) or off (repressor switch).

#### Activator switches

Variances of signaling times propagate additively through linear chains of genes in which each gene up-regulates the next. Let 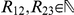 be threshold values for protein 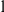 acting on promoter 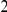 and protein 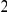 acting on promoter 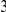, respectively. We assume that gene 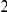 is switched on at time
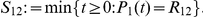



Analogously, let 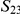 denote the length of time between 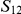 and the time at which the 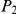 process first reaches level 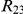. The distributions of 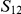 and 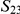 have PDFs of the form given in (18). Since 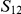 and 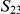 are independent, we have
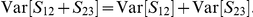



This argument extends inductively to directed pathways in which the product of each gene activates the subsequent gene in the sequence.

#### Repressor switches

Suppose that 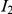 is on until time 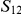, at which point 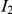 switches off. Queueing system 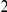 now has modified input rate function 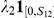, where 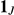 is the characteristic function of the interval 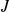. We compute 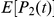 for 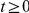 by conditioning on 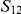. Let 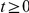. We have
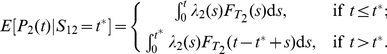
(23)Therefore
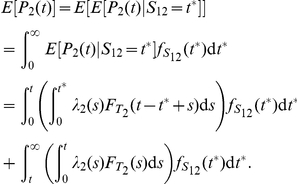
Higher moments may be obtained in a similar manner.

For a repressor switch, the 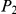 process and therefore the ability of gene 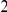 to signal downstream components depend in complex ways on the statistical properties of 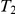. We examine these complex relationships by conditioning first on 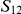 and then on 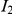. Suppose that 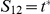. The key observation is this: for fixed 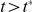, 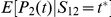 can *increase or decrease* with the standard deviation 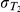 of 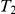. We verify this assuming 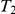 is symmetrically distributed about its mean and assuming 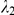 is a constant function.

If the midpoint 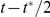 of the time interval 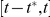 satisfies 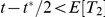, then
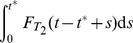
(24)is an increasing function of 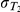 and therefore 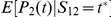 *increases* as 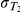 increases. By contrast, if 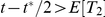, then the integral in (24) is a decreasing function of 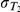 and therefore 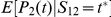 *decreases* as 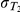 increases. Repressive signaling can therefore qualitatively affect the response of 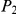 production to changes in the variability of 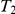.

**Figure 7:**
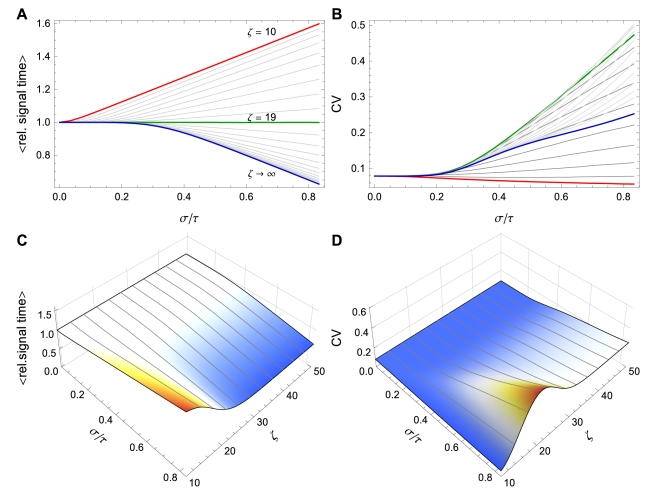
Signaling time depends on the number of initiation events. 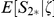
 can *increase or decrease* as a function of 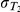 depending on the value of 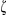. Here 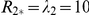. (A) 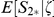 vs. CV of 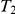 for 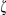 varying from 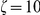 (red) to 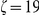 (green) to 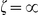 (blue) using the Bernoulli delay distribution 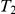 in Example with 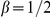. Note the transition that occurs at 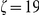. (B) Equivalent to (A), but plotting CV of the signaling time instead of conditional expectation. (C) and (D) Contour plots corresponding to (A) and (B), respectively. Notice that for fixed 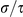, signaling time CV can change non-monotonically with 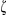. For instance, at 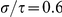, signaling time CV starts low (red), increases to 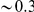 (green) and then decreases thereafter. Plots were obtained through stochastic simulation with 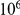 trials.

We now examine the ability of gene 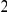 to signal downstream components by conditioning on 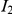. Let 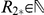. Let 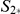 denote the time at which 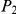 first reaches level 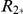. The key observation is this: If we assume that gene 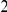 shuts off after exactly 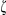 transcription initiation events, then 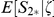 can *increase or decrease* as a function of 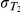. Figure 7 demonstrates this numerically for a case in which 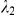 is a constant function and 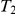 is symmetrically distributed. In this case, we find that 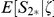

1. increases as 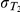 increases if 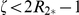,
2. does not depend on 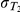 if 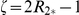, and
3. decreases as 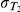 increases if 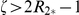.

Intuitively, this is due to the fact that the order in which proteins enter the queue is not necessarily the same as the order in which they exit. Consider again Figure 7. When the total number of transcription initiation events, 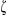, is smaller than 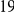, then more proteins enter the queue before the 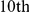 protein than after it. It is therefore more likely that a protein entering before protein 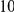 will exit ahead of it than that a protein entering after protein 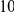 will exit before it. As a result, the expected time 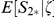 increases with 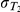. When the balance favors proteins that enter the queue after protein 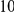, the opposite is true, and 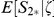 decreases with 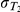.

We conjecture that this trichotomy holds in general if 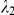 is a constant function and 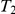 is symmetrically distributed about its mean.

### Stochastic simulations

Gillespie's stochastic simulation algorithm generates an exact stochastic realization for a system of 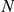 species interacting through 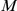 reactions. The state of the system is stored in the vector 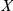, and each reaction 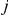 is characterized by a state change vector 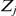 and its propensity function 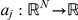. If the system is in state 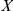 and reaction 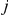 occurs then the system state changes to 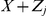 [5].

The idea behind extending Gillespie's SSA to model distributed delay is that if a reaction is to be delayed by some amount of time then we temporarily store this reaction along with the time at which the event will occur and we only apply this reaction at the given time. We used a version of the algorithm equivalent to those described in [32], [48]. Note that [48] also describes a more efficient version of the algorithm.

## References

[1] Jacob F, Monod J (1961). Genetic regulatory mechanisms in synthesis of proteins.. J Mol Biol.

[2] Alon U (2007). Network motifs: theory and experimental approaches.. Nat Rev Genet.

[3] De Jong H (2002). Modeling and simulation of genetic regulatory systems: a literature review.. J Comp Biol.

[4] Kærn M, Blake WJ, Collins JJ (2003). The engineering of gene regulatory networks.. Annu Rev Biomed Eng.

[5] Gillespie DT (1977). Exact stochastic simulation of coupled chemical reactions.. J Phys Chem.

[6] Kepler TB, Elston TC (2001). Stochasticity in transcriptional regulation: origins consequences, and mathematical representations.. Biophys J.

[7] Bundschuh R, Hayot F, Jayaprakash C (2003). Fluctuations and slow variables in genetic networks.. Biophys J.

[8] Bennett MR, Volfson D, Tsimring L, Hasty J (2007). Transient dynamics of genetic regulatory networks.. Biophys J.

[9] Lan YH, Elston TC, Papoian GA (2008). Elimination of fast variables in chemical Langevin equations.. J Chem Phys.

[10] McAdams HH, Shapiro L (1995). Circuit simulation of genetic networks.. Science.

[11] Goodwin BC (1965). Oscillatory behavior in enzymatic control processes.. Adv Enzyme Regul.

[12] Mahaffy JM, Pao CV (1984). Models of genetic control by repression with time delays and spatial effects.. J Math Biol.

[13] Chen L, Aihara K (2002). Stability of genetic regulatory networks with time delay.. IEEE Trans Circ Syst.

[14] Smolen P, Baxter DA, Byrne JH (1999). Effects of macromolecular transport and stochastic uctuations on dynamics of genetic regulatory systems.. Am J Physiol Cell Physiol.

[15] Monk NA (2003). Oscillatory expression of Hes1, p53, and NF–*κ*B driven by transcriptional time delays.. Curr Biol.

[16] Lewis J (2003). Autoinhibition with transcriptional delay: A simple mechanism for the zebrafish somitogeneis oscillator.. Curr Biol.

[17] Mather W, Bennett MR, Hasty J, Tsimring LS (2009). Delay-induced degrade-and-fire oscillations in small genetic circuits.. Phys Rev Lett.

[18] Amir A, Meshner S, Beatus T, Stavans J (2010). Damped oscillations in the adaptive response of the iron homeostasis network in *E. coli*.. Molec Microbiol.

[19] Stricker J, Cookson S, Bennett MR, Mather WH, Tsimring LS (2008). A fast, robust and tunable synthetic gene oscillator.. Nature.

[20] Tigges M, Marquez-Lago TT, Stelling J, Fussenegger M (2009). A tunable synthetic mammalian oscillator.. Nature.

[21] Larson DR, Zenklusen D, Wu B, Chao JA, Singer RH (2011). Real-time observation of transcription initiation and elongation on an endogenous yeast gene.. Science.

[22] Smolen P, Baxter DA, Byrne JH (2002). A reduced model clarifies the role of feedback loops and time delays in the drosophila circadian oscillator.. Biophys J.

[23] Sriram K, Gopinathan MS (2004). A two variable delay model for the circadian rhythm of neurospora crassa.. J Theor Biol.

[24] Ukai-Tadenuma M, Yamada RG, Xu H, Ripperger JA, Liu AC (2011). Delay in feedback repression by Cryptochrome 1 is required for circadian clock function.. Cell.

[25] Chen KC, Csikasz-Nagy A, Gyorffy B, Val J, Novak B (2000). Kinetic analysis of a molecular model of the budding yeast cell cycle.. Molec Biol Cell.

[26] Sevim V, Gong XW, Socolar JES (2010). Reliability of transcriptional cycles and the yeast cell-cycle oscillator.. PLoS Comp Biol.

[27] Tiana G, Jensen MH, Sneppen K (2002). Time delay as a key to apoptosis induction in the p53 network.. Eur Phys J.

[28] Bratsun D, Volfson D, Tsimring LS, Hasty J (2005). Delay-induced stochastic oscillations in gene regulation.. Proc Natl Acad Sci U S A.

[29] Maithreye R, Sarkar RR, Parnaik V, Sinha S (2008). Delay-induced transient increase and heterogeneity in gene expression in negatively auto-regulated gene circuits.. PLoS One.

[30] Scott M (2009). Long delay times in reaction rates increase intrinsic uctuations.. Phys Rev E.

[31] Grönlund A, Lötstedt P, Elf J (2010). Costs and constraints from time-delayed feedback in small regulatory motifs.. Proc Natl Acad Sci U S A.

[32] Schlicht R, Winkler G (2008). A delay stochastic process with applications in molecular biology.. J Math Biol.

[33] Arazi A, Ben-Jacob E, Yechiali U (2004). Bridging genetic networks and queueing theory.. Physica A.

[34] Levine E, Hwa T (2007). Stochastic uctuations in metabolic pathways.. Proc Natl Acad Sci USA.

[35] Mather WH, Cookson NA, Hasty J, Tsimring LS, Williams RJ (2010). Correlation resonance by coupled enzymatic processing.. Biophys J.

[36] Zhu R, Ribeiro AS, Salahub D, Kauffman SA (2007). Studying genetic regulatory networks at the molecular level: Delayed reaction stochastic models.. J Theor Biol.

[37] Zhu R, Salahub D (2008). Delay stochastic simulation of single-gene expression reveals detailed relationship between protein noise and mean abundance.. FEBS Lett.

[38] Bel G, Munsky M, Nemenman I (2010). The simplicity of completion time distributions for common complex biochemical processes.. Phys Biol.

[39] Elowitz MB, Levine AJ, Siggia ED, Swain PS (2002). Stochastic gene expression in a single cell.. Science.

[40] Gross D, Shortle J, Thompson J, Harris C (2008). Fundamentals of queueing theory. 4th edition.

[41] Thattai M, van Oudenaarden A (2002). Attenuation of noise in ultrasensitive signaling cascades.. Biophys J.

[42] Hooshangi S, Thiberge S, Weiss R (2005). Ultrasensitivity and noise propogation in a synthetic transcriptional cascade.. Proc Natl Acad Sci U S A.

[43] Mangan S, Alon U (2003). Structure and function of the feed-forward loop network motif.. Proc Natl Acad Sci U S A.

[44] Mangan S, Itzkovitz S, Zaslaver A, Alon U (2006). The incoherent feed-forward loop accelerates the response-time of the *gal* system in *escherichia coli*.. J Mol Biol.

[45] Goentoro L, Shoval O, Kirschner MW, Alon U (2009). The incoherent feedforward loop can provide fold-change detection in gene regulation.. Mol Cell.

[46] Amir A, Kobiler O, Rokney A, Oppenheim AB, Stavans J (2007). Noise in timing and precision of gene activities in a genetic cascade.. Mol Syst Biol.

[47] Golding I, Paulsson J, Zawilski SM, Cox EC (2005). Real-time kinetics of gene activity in individual bacteria.. Cell.

[48] Cai X (2007). Exact stochastic simulation of coupled chemical reactions with delays.. J Chem Phys.

